# Microvessel Density Is Associated with VEGF and α-SMA Expression in Different Regions of Human Gastrointestinal Carcinomas

**DOI:** 10.3390/cancers3033405

**Published:** 2011-08-31

**Authors:** Paola Tonino, Carmen Abreu

**Affiliations:** 1 Centro de Microscopía Electrónica “Dr. Mitsuo Ogura”, Facultad de Ciencias, Universidad Central de Venezuela, Apartado 76963, El Marqués 1070, Caracas, Venezuela; 2 Instituto Anatomopatológico, Facultad de Medicina, Universidad Central de Venezuela, Caracas, Venezuela; E-Mail: abreuc@med.ucv.ve

**Keywords:** MVD, VEGF, α-SMA, center of the tumor, periphery, metastasis, gastrointestinal cancer, clinicopathological factors

## Abstract

Tumor angiogenesis is known to be regulated by growth factors secreted by host and tumor cells. Despite the importance of tumor vasculature and angiogenic heterogeneity in solid tumors, few studies have compared the vasculature in different regions of human cancer. Blood vessels from different regions of carcinomas might have morphofunctional implications in tumor angiogenesis. In the present study, therefore, we have examined the relationship between microvascular density (MVD) and vascular endothelial growth factor (VEGF) expression and alpha smooth muscle actin (α-SMA) expression in the center of the tumor (CT), periphery (P) and metastasis (M) regions from gastrointestinal carcinomas (GITC), as well as the association of MVD with clinicopathological factors. Surgically resected specimens corresponding to the CT, P and M from 27 patients were examined for FVIII, VEGF and α-SMA by immunohistochemistry. The MVD was not significantly different in the CT, P and M regions from GITC. The MVD in the VEGF positive group was significantly higher than in the VEGF negative group (CT, p = 0.034; P, p = 0.030; M, p = 0.032). The MVD as a function of α-SMA expression was also significantly higher in the CT and P region compared to the M region (p = 0.0008). In conclusion, the MVD association with VEGF and α-SMA expression, might indicate an increase of the number of neoformed and preexisting blood vessels uniformly or partially covered by pericytes in different regions of GITC, suggesting that not only MVD and VEGF are important parameters to the tumor vasculature, but also blood vessels maturation is a crucial factor for gastrointestinal tumor angiogenesis regulation and possible target of vascular therapy.

## 1. Introduction

The importance of tumor angiogenesis in the growth, progression and metastasis of solid tumors is widely known. Blood vessels are recruited by tumors from the neighboring tissues by this process, in which a plethora of angiogenic factors are involved [1]. Among them, the vascular endothelial growth factor (VEGF) is one of the most critical proteins to influence the angiotumoral dynamic with cell signaling responses in proliferation and metastasis [2]. It is known that the tumor growth and metastasis not only depend on distinct biological properties of cancer cells, but also in multiple and complex tumor microenvironment interaction with the host cells and factors that maintain the tissues homeostasis [3].

Since the last decade translational cancer biology has evolved with the prolific research addressing the clinical implications of angiogenic response, quantitated or in relation with prognosis, and the role of inhibitory molecules/factors to slow the tumor proliferation. Furthermore, the blood vessels remarkable features and diverse biomarkers expression in tumors have been crucial for the development of new vascular therapies [3]. The number and distribution of blood vessels in different regions of tumors might also be relevant for tumor growth and metastasis, delivery of anti-neoplastic drugs, radiotherapy effectiveness, clinic outcome and prognosis [4,5]. Thus, the increase in the microvascular density (MVD) has been usually observed in the periphery of the tumor, whereas the center of the tumor is characterized by a decreased MVD, dilated vessels and necrosis [6]. In the last two decades, the increased MVD has been associated with early progression in several tumors including the gastrointestinal cancer [7].

Much of the evidence for tumor dependence on angiogenesis is based on animal studies, in which qualitative analysis have shown that solid tumors are less vascular in their center and host tissues become more vascular in close proximity to the invasive edge of tumors [5,8]. However, the malignancies that arise in the human gastrointestinal tract reveal some significant differences from rapidly growing experimental models, whose kinetics are very different from the growth kinetics of human tumors [2,9]. Despite the importance of the angiogenic heterogeneity in solid tumors, mostly confirmed in experimental models, the relationship between MVD, angiogenic factors expression and vascular maturity in different regions of human gastrointestinal cancer has not been clarified. In the present study, therefore, we have examined the relationship between MVD and VEGF and α-SMA expression in the center of the tumor (CT), periphery (P) and metastasis (M) regions from gastrointestinal carcinomas (GITC), as well as the correlation of MVD with clinicopathological findings in these tumors.

## 2. Results

### 2.1. Microvascular Density and Correlation with Clinicopathological Factors

**Figure 1.**
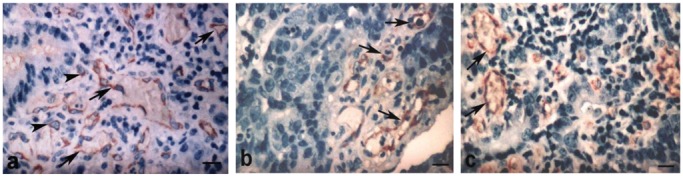
Representative FVIII immunostaining in blood vessels (arrows) and sprouts (arrowheads) in the center of the tumor (a), periphery (b) and metastasis (c) regions from GITC. Blood vessels with variable diameters and infiltration of tumor cells into lumen were also observed in the periphery region. Bar = 25 μm.

The FVIII expression (CT, 76.62%; P, 100%; M, 71.42%) was observed in the cytoplasm of endothelial cells and sprouts, illustrated in Figure 1.

The MVD in different regions from GITC was increased in the stroma and in the proximity of tumor parenchyma. The heterogeneity of MVD was observed not only in different tumors, but in distinct regions of the same tumor. The MVD mean ± SEM in most active areas of neovascularization from CT, P and M regions was 28.78 ± 3.67 blood vessels/mm^2^ (range, 3-116; median 22.00); 28.44 ± 2.84 blood vessels/mm^2^ (range, 3–102; median, 20.00) and 24.12 ± 2.83 blood vessels/mm^2^ (range, 3–62; median, 22.00), respectively. Although the MVD was not significantly different in the CT, P and M regions, it was closely related to the tumor localization, tumor size and histological degree differentiation, but not associated with the lymph nodes metastasis (Table 1).

### 2.2. VEGF Expression and Relationship with MVD

**Figure 2.**
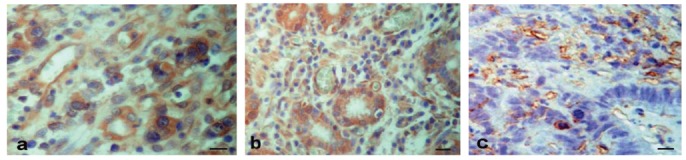
Representative sections of GITC immunohistochemically stained with VEGF in tumor cells and blood vessels in the center of the tumor (**a**), periphery (**b**) and metastasis (**c**) regions. Note the nuclear staining in tumor cells and the presence of blood vessels in the proximity of tumor glands in the periphery region. In (a) and (c), bar = 25 μm and in (b), bar = 35 μm.

The immunohistochemical analysis of VEGF expression, representative in Figure 2, showed positive staining (CT, 84.6%; P, 100%; M, 80%) in the cytoplasm or the nucleus of tumor cells, in endothelial cells and in the stromal cells. VEGF expression in tumor cells was predominantly moderate in the CT and M regions, and frequently strong in the P region. The nuclear expression of VEGF in tumor cells from CT and P regions was also confirmed at ultrastructural level (data not shown).

The MVD as a function of the expression of VEGF in GITC was significantly higher in the CT region of those tumors with VEGF expression of 1 compared to the MVD in the P region (p = 0.013) and M region (p= 0.000007). MVD was not significantly different between those tumor regions with VEGF expression of 2. However, MVD was significantly higher in the CT region in those tumors with VEGF expression of 3 compared to the MVD in the P region (p = 0.008). MVD in the VEGF positive group was higher than in the negative group (CT, p = 0.034; P, p = 0.030; M, p = 0.032) as is shown in Table 2.

### 2.3. α-SMA Expression in Periendothelial Cells and Association with MVD

**Figure 3.**
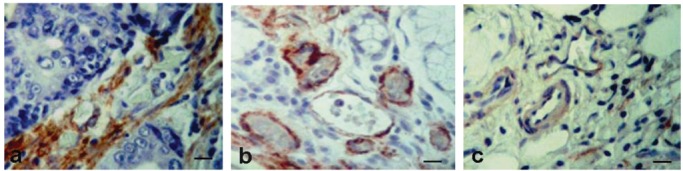
Immunohistochemical staining of α-SMA in periendothelial cells surrounding blood vessels in the center of the tumor (**a**), periphery (**b**) and metastasis (**c**) regions from GITC. α-SMA expression was observed in a continuous and discontinuous pattern of distribution around blood vessels. Bar = 25 μm.

In different regions of GITC, the expression of α-SMA was observed in pericytes surrounding the blood vessels continuously distributed or in a discontinuous pattern, as is shown in Figure 3. The stroma cells surrounding or in the proximity of tumor cells were also α-SMA+. The coverage of blood vessels with pericytes α-SMA+ was 12.71% in the CT, 29.88% in the P region, and 15.17% in the M region. The proportion of blood vessels covered by pericytes α-SMA+ uniformly distributed was higher in the P region (82.36%) compared to the CT (45.46%) and M region (59.10%). These results indicate that the tumor vasculature of GITC is composed by a high proportion of blood vessels covered uniformly by pericytes α-SMA+ predominantly in the P region, as well as blood vessels with pericytes α-SMA+ discontinuously distributed in the CT and M regions.

**Figure 4.**
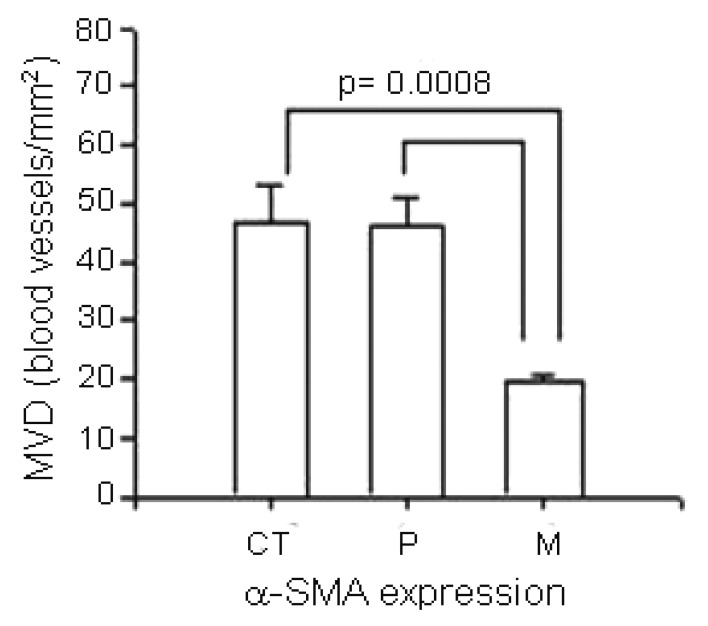
Microvascular density as a function of α-SMA expression in periendothelial cells from different regions of GITC. Data are presented as mean ± SEM (differences between center of the tumor, periphery and metastasis regions: *p < 0.05).

The relationship between MVD and α-SMA+ expression is graphically illustrated in Figure 4. The MVD in the CT and P regions was significantly higher compared to the MVD in the M region (p = 0.0008).

### 2.4. Discussion

In our study, we showed an association between MVD and VEGF and vascular maturity through α-SMA expression in different regions of GITC. MVD was also correlated with several pathological parameters such as tumor size, tumor location and histological degree differentiation. The quantification of MVD, although assess the presence of blood vessels is not sufficient to reveal the functional or angiogenic status of tumor vasculature, but it reflects the metabolic burden of the supported tumor cells and the intercapillary distance [10]. In general, the MVD has been quantified by immunohistochemical staining of tumor blood vessels with the use of antibodies, including CD31, CD34, von Willebrand Factor (vWF) or Factor VIII-related antigen (FVIII) and CD105. The vWF is a large multimeric glycoprotein synthesized exclusively in endothelial cells and megakaryocytes, stored in the Weibel-Palade bodies in the cytoplasm of endothelial cells and in platelet α-granules, respectively (for a review see [11]). We used the polyclonal anti-FVIII antibody for the MVD assessment, whose reproducibility of blood vessels immunostaining in different regions from GITC was higher compared to the anti-CD31/PECAM antibody immunostaining (data not shown). These results were in concordance with other comparative studies of MVD that used these antibodies in breast, prostate, gastric, esophageal and lung cancer [3,12,13]. The FVIII expression observed in endothelial cells cytoplasm corresponding to sprouts in the CT and P regions from GITC could indicate the occurrence of early angiogenesis events in these tumors. The increased MVD in the stroma of the CT, P and M regions could be related to the secretion of angiogenic factors by tumor cells in a short distance and the recruitment of blood vessels around the tumor cells [8]. Moreover, the heterogeneity of MVD was observed not only in different tumors, but in distinct regions of the same tumor, similar to previous findings in colorectal cancer [14,15]. Although in our results the MVD was not significantly different in the three regions analyzed with the anti-FVIII antibody, the CT region was an active area of blood vessels with expression of angiogenic factors, such as VEGF and angiopoietins (data not shown), which promotes neovascularization and also coexists with the process of necrosis and apoptosis. These results are in good agreement with previous studies on colorectal cancer not significantly different between the MVD of central and peripheral regions [2]. It has been suggested that tumor growth might be transformed into a compartmentalized network with irregular dilated vessels and a decreasing MVD from the tumor periphery to the tumor center that causes an inhomogeneous oxygen distribution [16]. Nevertheless, in GITC the MVD do not decrease from the P region to the CT region or even in metastasis tissue, possibly related to an increased expression of VEGF as has been observed in these tumors. It is also widely known that more than one factor is required to evoke an angiogenic response, because tumor cells under hypoxia secrete increased amounts of growth factors that bind to their specific receptors in the surface of endothelial cells and stimulate the formation of new blood vessels.

The correlation of MVD with several clinicopathological factors in different regions of GITC was similar to the findings in oral squamous cell carcinoma [17] However, we did not find an association between MVD and the lymph nodes metastasis, corroborating other reports on gastric and colorectal carcinomas [12,14,18,19]. On the contrary, several studies have shown that the MVD and lymph node metastasis are independent prognostic factors in esophageal, gastric and colorectal carcinomas [14,15,20-22], but not related to the tumor size, histological degree of differentiation or tumor location. Recently, it has been reported that MVD measured with CD34 antibody is related to advanced gastric cancer, the risk of upper gastrointestinal bleeding and survival rate [23]. In this study the mean MVD was lower than our MVD values from different regions of GITC. This controversy may be a result from several factors, such as the number of patients (clinical samples) considered in each study, the tissue heterogeneity, the standardized area of tumor sampling (hot spots), and the methodological variability regarding the use of pan-endothelial markers and specific antibodies to activated or proliferating endothelium for the blood vessels detection, as well as whether MVD was assessed in both, peritumoral and intratumoral tissues.

It is widely accepted that the tumor vascularization depends on the balance of agonist and antagonist factors of angiogenesis released by tumor cells and host cells, which stimulate or inhibit the neovasculature, respectively [24]. In cancer tissue, the VEGF has been involved in multifunctional dependent and independent roles of endothelial cell, upregulated by oncogenes activation [25], lost of tumor suppression [26,27], activation of growth factors/cytokines [28] and induction of hypoxia or hypoglycemia [29].

The immunohistochemical analysis of VEGF showed an increased angiotumoral expression in different regions of GITC, similar to the previous observations in a number of malignant tumors, including those arising in the gastrointestinal tract [30]. The moderate to strong VEGF expression in GITC was significantly associated with a high MVD, in agreement with the findings in esophageal and gastric cancer and colorectal carcinoma [18,20]. The VEGF expression in the M region was possibly linked to an accumulation of the protein instead of synthesis. This observation was previously reported in Hodking disease [31]. The VEGF expression into the necrosis area is probably related to the hypoxia typical of tumors, as has been reported in esophageal and gastric carcinoma [20].

The MVD association with VEGF expression was in concordance with the increase of blood vessels in the CT, P and M regions from GITC with positive VEGF immunoreactions, confirming that these tumors increase their vasculature at expenses of at least VEGF. This growth factor is associated with tumor cell proliferation as previously reported [27]. Our results are also in line with findings in gastric and esophageal cancer [12,20,32], stromal tumor of stomach [33] and colorectal cancer [18,34], in which the VEGF expression and MVD have been considered as prognostic factors. In a previous study we also showed that intratumoral VEGF expression in GITC is correlated with the tumor size, infiltration, vascular invasion, and interestingly gastritis, suggesting an important association with a poor prognosis [27].

The VEGF influence on angiogenesis depends on tumor cell expression and its binding to VEGF receptors (VEGFRs) [7]. The signaling pathway of VEGF initiate the neovascularization by the recruitment of endothelial cells conducting the capillary tube formation and stabilizing it following a sequence of molecular events that involve mural cells (pericytes and smooth muscle cells) through the plaquets derived growth factor (PDGF), binding to VEGFRs, and extracellular matrix generation [35]. VEGF has also been involved in pericyte recruitment to form functional vascular networks in physiological and in pathological conditions [36]. The morphofunctional characteristics of pericytes determine their roles in tumor angiogenesis. In recent years, these cells have been considered as potential new targets for antiangiogenic therapies [37]. The presence of pericytes and smooth muscle cells surrounding blood vessels has been described as a structural parameter indicative of vascular maturity. Pericytes are known to express several biomarkers such as, α-SMA, the PDGF receptor (PDGFR-β), and desmin [38,39]. Moreover, both pericytes and smooth muscle cells can also be identified in the vasculature with the proteoglycan NG2, the aminopeptidase N and the expression of XlacZ gene [39]. Interestingly, the α-SMA monoclonal antibody has also been used to immunostain the pericytes in myelofibrotic bone marrows “liquid tumors”, since in most tissues this marker has been found reliably to identify these cells, and also due to the variable specificity with antibodies to desmin or PDGFR-β [40]. Besides, the immunohistochemical and structural properties of pericytes have been related to several factors including, the tumor grade, histological differentiation, stage, location, age, treatment, as well as the conditions of the tissue fixation for the analysis [41].

The pericytes coverage of vessels is considered an important factor involved in the abnormalities of tumor vasculature [41]. In general, tumor blood vessels are often characterized by decreased pericyte coverage, making them less stabilized, more permeable and more sensitive to angiogenic stimuli with variations among capillary beds of different tissues [42]. In our study, the coverage of pericytes α-SMA+ was higher in the P region compared to the CT and M regions. The decrease in the expression of α-SMA in cells surrounding the vasculature of GITC in the CT and M regions might suggest that blood vessels are fragile and immature, as has been observed in malignant melanoma [43]. The variation in the recruitment of pericytes in blood vessels from GITC could be a sign of vascular maturation/remodelation in these tumors previously reported in colon, breast, lung and prostate cancer (41). Likewise, the higher expression of α-SMA in a continuous or uniform pattern of distribution in the pericytes of blood vessels from the P region compared to the CT and M regions could indicate either the heterogeneity of pericytes in GITC or the gradients of α-SMA expression with influence in its function [43]. These results are similar to the findings in the vasculature of transgenic mice (RIP-Tag2) inoculated with tumor cells from insulinoma, and singenic mice implanted with MCa-IV cell lines from breast cancer and Lewis lung cancer, where the abnormal pattern of α-SMA expression has been associated with structural alterations of blood vessels [38]. More recently, the increased pericytes coverage of blood vessels was associated with alterations of the vascular morphology in patients with myelofibrosis [40].

A diverse degree of neovasculature maturation has been associated to the recruitment of mural or periendothelial cells α-SMA+. The mature phenotype of the quiescent vasculature in organs is mainly characterized by the extent recruitment of pericytes as a possible control mechanism of quiescent endothelial cells phenotype [41]. It is also known that the interaction between pericytes and endothelial cells is essential to the surveillance, stability and maturation of endothelial cells through the paracrine signaling pathway of both, PDGFB and PDGFR-β expressed in endothelial cells and pericytes, respectively [44]. In prostate cancer and glioblastoma, a fraction of angiogenesis involves immature vessels with the absence of associated pericytes or smooth muscle cells that express α-SMA, which are selectively obliterated by VEGF withdrawal [45]. Additionally, it has been suggested that the lack of the proper arrangement of periendothelial cells in tumor vessels might contribute to the abnormal phenotype of tumor vasculature characterized by an irregular structure and inefficient blood flow, which depend on a continuous supply of VEGF [46]. Besides, the insufficient pericyte coverage has also been involved in the overexpression of VEGF since pericytes not only act to stabilize microvessels, but function to provide local blood flow [7]. In our study, the insufficient pericyte coverage in the CT and M regions possibly contribute to insufficient blood flow, resulting in the overexpression of VEGF [36].

Mature vessels are less leaky and morphofunctionally characterized by quiescent, differentiated and functional networks, which involve suppression of endothelial cells proliferation, protection against VEGF withdrawal, stabilization of vascular tubes, fenestrations and tight junction barriers, as well as the recruitment of mural cells (pericytes and smooth muscle cells) into the vessel wall. These features might have an indirect impact on reducing tumor angiogenesis and improvement in survival of cancer patients. Recently, Goel *et al.* [47] reviewed that anti-VEGF therapy in preclinical studies caused changes in the tumor vasculature towards a more “mature” or “normal” phenotype characterized by attenuation of hyperpermeability, increased vascular pericyte coverage, a more normal basement membrane, and a reduction in tumor hypoxia and interstitial fluid pressure. Thus, the impact of these changes are reflected in the improvement in the metabolic profile of the tumor microenvironment, the delivery and efficacy of cytotoxic drugs to tumor cells, the efficacy of radiotherapy and the effectors immune cells (e.g., T lymphocytes). Interestingly, Piña *et al.* [48] suggested that the heterogeneity and spatial distribution of the tumor vasculature in retinoblastomas is clinically significant since blood vessel maturation may limit antiangiogenic therapies that mainly target immature vasculature.

The presence of stromal cells α-SMA+ observed in peritumoral and intratumoral GITC was considered as myofibroblasts abundantly present in several tumors but not in normal tissue, and might be related to stroma formation as previously observed in gastric carcinoma [49].

A positive significant correlation between MVD and α-SMA expression in the CT and P region compared to the M region of GITC was similar to the findings in breast in situ carcinoma [50], suggesting that in those regions a proportion of blood vessels are mature and might contribute to the gastrointestinal tumor angiogenesis and the vascular remodelation.

In conclusion, the MVD association with VEGF and α-SMA expression, might indicate an increase of the number of both neoformed and preexisting blood vessels, either uniformly or partially covered by pericytes in different regions of GITC, suggesting that not only MVD and VEGF are important parameters to the tumor vasculature, but also blood vessels maturation is a crucial factor for gastrointestinal tumor angiogenesis regulation and vascular therapy.

## 3. Experimental

### 3.1. Patients and Samples

Specimens from different tumor regions from GITC were surgically removed and collected from March 1997 to February 2002 from patients (n= 27; 19 males and 8 females), 44 to 76 years (mean age of 67 years) and a histopathological diagnosis of GITC (n= 4 stomach; n= 19 colon, and n= 4 rectum). No patient in this study was treated with chemotherapy or radiotherapy prior surgery. The regions are defined as: CT-all tumor mass except for peripheral tissue immediately adjacent to the invasive edge; P-peripheral tissue of the tumor immediately adjacent to the invasive edge, and M-all tumor tissue from lymph nodes metastasis. The study was approved by the local ethic committee from the Hospital Oncológico Padre Machado and the Hospital Clínico Universitario de Caracas, Universidad Central de Venezuela.

### 3.2. Immunohistochemistry for FVIII, VEGF and α-SMA Expression in the Vasculature

Paraffin-embedded sections from CT, P and M region tissues were processed for immunohistochemical analysis as previously described [27]. In brief, endogenous peroxidase blocking and proteolytic predigestion when needed were performed with 3% hydrogen peroxide in methanol for 5 min and 0.1% Trypsin (Lipshaw Immunon, Pittsburgh, PA, USA) in Tris buffer (150 mM, 3.3 mM Ca2Cl, pH 7.6) for 5 min at 37 °C, respectively. The blocking reagent from labeled streptavidine biotine (LSAB) kit (DAKO, Carpinteria, CA, USA) was used to reduce nonspecific antibody binding. The sections were then incubated with rabbit polyclonal anti-FVIII (A0082; 1:300 dilution) (DAKO), rabbit polyclonal anti-VEGF (A-20; 1:300 dilution) (Santa Cruz Biotechnology, Santa Cruz, CA, USA) and mouse monoclonal anti-α-SMA (M0851; 1:35 dilution) (DAKO) antibodies. After incubation with biotinylated secondary antibodies from LSAB kit for 30 min, and then with streptavidin-peroxidase reagent from this kit for 30 min according to the manufacturer's instructions, the immunoreactions were developed with 3-amino-9-ethyl carbazole (Sigma Chemical Co., St Louis, MO, USA) solution, counterstained with Mayer hematoxylin, and mounted in aqueous medium. Negative controls for each tissue section were prepared replacing the primary antibody with PBS. Controls consisted of normal gastrointestinal mucosa tissues.

The intensity of VEGF immunostaining was scored as negative= 0, weak= 1, moderate= 2 and high= 3. The proportion of blood vessels with periendothelial cells α-SMA-positive (α-SMA+) expression was defined as the ratio between the number of blood vessels with periendothelial cells α-SMA+/mm^2^ and the number of blood vessels FVIII+/mm^2^ × 100 at a magnification of 40X (area 0.159 mm^2^). Observations were performed at the microscope standard KF2 Zeiss with a digital camera (Casio QV-R40). The immunohistochemical evaluation of the specimens was performed on coded samples by two observers independently, without knowledge of the clinicopathological factors.

### 3.3. Microvascular Density Assessment

MVD (number of blood vessels/mm^2^) was determined in sections from CT, P and M region tissues immunostained with FVIII according to the Weidner *et al.* method [5]. Sections were observed at low magnification to identify the hot spots areas in different regions from GITC. The MVD was quantified in 5 fields of 250× (area of 0.639 mm^2^) and 400× (area of 0.159 mm^2^) and values were expressed as the mean ± SEM.

### 3.4. Statistical Analysis

The Student's *t* test were used to compare the MVD in CT, P and M region from GITC, as well as to examine the relationship between MVD and VEGF expression or α-SMA expression. The association between MVD and clinicopathological variables was examined by ANOVA. The statistical significance was defined as p < 0.05. All data were analyzed using KaleidaGraph v. 3.6 software.

## 4. Conclusions

The MVD was significantly associated with VEGF and α-SMA expression and related to tumor location, tumor size and histological degree of differentiation; however the MVD was not significantly different in the CT, P and M regions from GITC. The P region reflected the higher α-SMA+ pericytes coverage of blood vessels, mostly uniformly distributed compared to the CT and M regions, which also overexpressed VEGF in both, tumor cells and endothelial cells. Taken together these results suggest that MVD, VEGF and vascular maturity are related as important factors involved in gastrointestinal tumor biology, which suggest that future research should focus on different regions of the tumor, not only into the morphofunctional aspects of endothelial cells, but in the maturity of blood vessels through the presence of periendothelial cells, as possible targets of vascular therapy.

## Figures and Tables

**Table 1.** Relationship between clinicopathological factors and MVD.

**Mean MVD**					

**Variables**	**n (%)**	**CT**	**Regions P**	**M**	**p value**
Age					
>67	14	15.09	17.64	24.80	NS
<67	13	22.67	22.86	23.95	
Gender					
Male	13	21.86	28.60	33.50	NS
Female	14	29.15	28.26	27.86	
Tumor location					
Stomach	4	44.25	41.60	26.70	0.00001*
Colon	19	32.90	30.65	21.13	
Rectum	4	11.25	9.80	5.50	
Tumor size					
> 6	8	47.80	33.80	19.40	0.0082*
< 6	19	24.02	28.35	25.30	
Histology grade					
Differentiated	25	33.75	32.30	12,60	0.0002*
Undifferentiated	2	8.90	10.80	24,60	
Lymph nodes metastasis					
Positive	14	29.16	29.46	28.80	NS
Negative	13	29.00	27.51	17.10	

NS:non significant; ANOVA

* p < 0.05

**Table 2.** Relationship between MVD and VEGF expression in different regions of GITC.

**Region**	**VEGF expression**	**MDV (mean ± SEM)**	**p-value**
CT	-	12.9 ± 2.9	0.034*
	+	36.4 ± 6.5	
P	-	0.0	0.030*
	+	33.9 ± 3.1	
M	-	19.6 ± 0.2	0.032*
	+	25.5 ± 1.2	

CT: center of the tumor; P: periphery; and M: metastasis. Student's *t*-test statistically significant

* p < 0.05 *vs.* VEGF negative group.
